# Continued Malarial Fever

**Published:** 1898-07

**Authors:** W. B. Campbell

**Affiliations:** Chappell Hill, Texas


For the Texas Medical Journal.

### Continued Malarial Fever.

BY W. B. CAMPBELL, M. D., CHAPPELL HILL, TEXAS.

[Read before the Brenham Medical Society, April 5th, 1898.]

This is a subject which can justly lay claim to no small importance, and, I think, one that should receive especial attention. As the symptoms in *all* cases are not identical, a procedure that would suit *one* case would be much out of place in another.

True, we can group many of the symptoms and formulate a plan which would produce good results in the majority, but routine practice is very dwarfing to one's mental capacity,--if not to the dimensions of our cemeteries. *Each* case should be judged according to its own indications and treatment formulated accordingly. Continued malarial fever being carefully distinguished from its closely allied sisters, typhoid and remittent malaria and *treated* as remittent malarial fever and no other—though I am aware that many doubt the very existence of this form of fever. However, Loomis includes this fever in his list of malarial fevers, and although it is not altogether malarial in its origin, malarial poison is essential to its development. Continued malarial fever has, as I said before, many elements in common with typhoid and many with remittent. By some it has been called "typho-malarial"--a misnomer--the term being used to avoid confusion and to convey the idea of a septic and a malarial poison—not the bacillus of Eberth, for if such were the case, it would then be compelled to run the prescribed course of typhoid fever, and would in fact *be* typhoid fever. Although this fever is closely allied to typhoid and has many elements in common, still the temperature record of the first week, the tongue, characteristic diarrhoea and rose colored spots should prevent any error in the diagnosis. The course of typhoid fever being marked—even and regular exascerbations and remissions—red pointed tongue, becoming dry, brown and cracked,—while on the other hand the temperature is variable, exascerbations and remissions not so well marked—sometimes there being no perceptible remissions in the early stage of the fever. The tongue is pale and flabby at first, and smooth; is soon covered with a yellowish white coat. As the fever progresses the tongue becomes red, the coat brownish. Sometimes red and shining with sordes collected on the teeth and lips—the coating on the tongue is sometimes thrown off in flakes, the tongue assuming a beefy red appearance, and in a short while, again becomes brown and dry with a renewal of the fever symptoms. Some regard the so called typhoid element as nothing more than the typhoid condition liable to be developed in connection with remittent fever as well as many other diseases. While we see that there are no pathological lesions that can be regarded as characteristic of this fever—and while the lesions very closely resembles those of typhoid fever and remittent fever—still its *clinical symptoms* are sufficient to stamp it as a *distinct* type of fever. It is difficult to ascertain the true cause of this fever. That *malarial* poison is necessary to its development, no one doubts. Though 'tis met with only in malarial districts, it is certain that wherever it prevails some other poison besides malaria is in operation.

This is not an infectious disease as is typhoid. In my limited experience I have never had more than one patient in a homestead, even though some member of the family was constantly present and in some instances even sleeping with the patient and remaining in perfect health. It is impossible to give an outline of the symptoms true to all or even the major portion of cases. In *some* cases there are no *premonitory* symptoms; in *others*, pain in the limbs and back, headache, exhaustion, loss of appetite preceed the chill. When once established the fever is variable—the first few days resembling closely simple remittent fever—though the *remissions* are never so well defined. As the disease *progresses* the exascerbations become shorter and the remissions longer, and at times a normal and *even subnormal* temperature is reached. Abdominal tenderness, especially in the right iliac fassa is not common, in fact is very seldom met with; soreness around umbilicus; throughout the whole course of the disease there is a marked tendency to periodicity. Diarrhœa is present at almost any stage of the fever. Often complications arise, bronchitis being the most common. In typhoid fever rose-colored spots appear on the chest and abdomen on the 6th to 8th day; in the other—although an eruption is *sometimes present*, it has *none of the characteristics of the typhoid element*—is not rose-colored, and does not dissappear on pressure but remains visible throughout the whole course of the disease. Besides the absence of these symptoms of typhoid fever there is, in continued malarial, a periodicity in the febrile action. In cases that recover, the average duration of the disease is from 3 to 4 weeks, convalescence being protracted. Statistics show 8 to 10 per cent mortality varying in the different localities in which it occurs, the *hygienic surroundings* and the *range of temperature* greatly influence the prognosis, as *also* whether the *malarial* or the *septic* element predominates, the *septic* type being more fatal. In those cases in which *malarial* symptoms predominate, *Quinine* will in many instances arrest its progress and shorten its duration, but in those cases in which the *septic* element predominates, while quinine may act as an antipyretic it has little if any power in arresting its progress or shortening its duration, though in many cases it renders the course of the fever *milder*. Usually I use a chlorine mixture, this acting as a *heart stimulant, antipyretic and intestinal antiseptic*; at the same time giving 5 grs. Quin. Sulph. every 2 hours until patient is *thoroughly* cinchonized, then discontinue quinine for a day or so, continuing chlorine mixture, *recinchonizing* my patient and so on throughout the course of the disease, using milk as the principal diet. In some cases I give sulph. cinchonidia with good results.

